# The complete chloroplast genome sequence of *Distylium macrophyllum* (Hamamelidaceae)

**DOI:** 10.1080/23802359.2019.1703606

**Published:** 2020-01-20

**Authors:** Shuifei Chen, Yao Li, Lu Wang, Lei Xie, Xiaomin Ge, Xu Zhou, Yaping Hu, Wenwen Zhang, Yanming Fang, Hui Ding

**Affiliations:** a Research Center for Nature Conservation and Biodiversity, State Environmental Protection Scientific Observation and Research Station for Ecology and Environment of Wuyi Mountains, State Environmental Protection Key Laboratory on Biosafety, Nanjing Institute of Environmental Sciences, Ministry of Ecology and Environment, Nanjing, China;; b Co-Innovation Center for Sustainable Forestry in Southern China, College of Biology and the Environment, Key Laboratory of State Forestry and Grassland Administration on Subtropical Forest Biodiversity Conservation, Nanjing Forestry University, Nanjing, China

**Keywords:** Chloroplast genome, *distylium macrophyllum*, hamamelidaceae, phylogeny

## Abstract

*Distylium macrophyllum* H.T.Chang is a critically endangered tree species endemic to southern China. In this study, we assembled and characterized the plastome of *D. macrophyllum* using Illumina paired-end data. The circular genome is 159,089 bp in size, consisting of two copies of inverted repeat (IR) regions of 26,235 bp, one large single-copy (LSC) region of 87,822 bp, and one small single-copy (SSC) region of 18,797 bp. It encodes a total of 114 unique genes, including 80 protein-coding genes, 30 tRNA genes, and four rRNA genes. Phylogenetic analysis based on 13 cp genome sequences indicated that *D. macrophyllum* was sister to the clade of *D. tsiangii* and *Parrotia subaequalis*.

*Distylium macrophyllum* H.T.Chang is an evergreen shrub or small tree endemic to southern China. It is mainly distributed in the mountainous areas of northern Guangdong and northwestern Guangxi (Chang 1960). Due to the extremely small population size and narrow range, the species has been classified as critically endangered in the Red List of China Higher Plants (Qin et al. 2017). During the fieldwork, we also found that some natural habitats of the species were degraded or lost due to urban and agricultural development, which would further increase the risk of wild extinction. However, since firstly described by Hung-ta Chang in 1960, there is limited research on the conservation genetics of the species. In this study, we report the complete chloroplast (cp) genome of *D. macrophyllum* based on Illumina pair-end sequencing data to provide more genomic resources for future conservation.

Fresh young leaves of *D. macrophyllum* were sampled from a wild individual growing at the Tielongtou Village, Ruyuan, Guangdong Province, China (25.020°N, 113.186°E). The voucher specimen (accession number 19090502) was preserved at the Herbarium of Nanjing Forestry University (HNFU). Total DNA extraction and whole genome sequencing on the Illumina Hiseq X Ten platform were conducted by Nanjing Genepioneer Biotechnologies Inc. (Nanjing, China). A total of 24,105,337 clean reads were produced and then used for the *de novo* assembly with NOVOplasty 2.7.2 (Dierckxsens et al. 2016). Gene annotation was performed using the CpGAVAS pipeline (Liu et al. 2012).

The complete cp genome of *D. macrophyllum* (GenBank accession number MN729500) is a circular molecule of 159,089 bp in length, consisting of two copies of IR (26,235 bp) separated by the LSC (87,822 bp) and SSC (18,797 bp) regions. The overall GC content was 38.01%, while the corresponding values of the LSC, SSC, and IR regions were 36.17%, 32.48%, and 43.06%, respectively. The cp genome encoded a total of 132 genes, of which 114 were unique and 18 were duplicated in the IR regions. The 114 unique genes contained 80 protein-coding genes, 30 tRNA genes, and four rRNA genes. Eighteen genes contained introns, 15 of which (nine protein-coding genes and six tRNA genes) contained one intron, and three of which (*rps12*, *ycf3*, and *clpP*) contained two introns.

A Bayesian phylogeny was reconstructed using cp genome sequences of 13 species in the woody Saxifragales to determine the phylogenetic position of *D. macrophyllum* (Figure 1). Two locally collinear blocks were identified and a matrix of 93,936 bp was generated by the HomBlocks pipeline (Bi et al. 2018). Using PartitionFinder 2 (Lanfear et al. 2016), the TVM + I + G model was chosen for the subset of LCBs 1-2. A Markov chain Monte Carlo (MCMC) as implemented in MrBayes 3.2.6 (Ronquist et al. 2012) was run for 1,000,000 generations with two parallel searches using four chains, each starting with a random tree. Trees were sampled every 100 generations and the first 25% was discarded as burn-in. Our results indicated that *D. macrophyllum* was sister to the clade of *D. tsiangii* and *Parrotia subaequalis* with strong support (posterior probability = 1.0).

**Figure 1.**
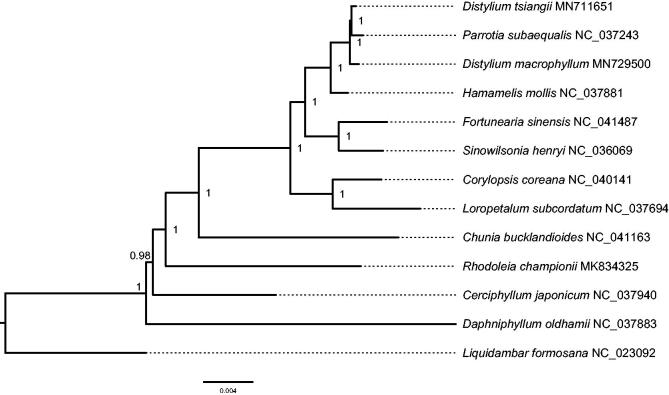
Bayesian phylogeny reconstructed by MrBayes 3.2.6 (Ronquist et al. 2012) using cp genome sequences of 13 species in the woody Saxifragales. The posterior probability value is labeled for each node.
